# Feedback-driven rule induction and retrieval-augmented bias mitigation for large language models

**DOI:** 10.3389/frai.2026.1925701

**Published:** 2026-09-22

**Authors:** Abhishek Kumar, Aishwaryaa Shree Muralitharan, Vinesh Kannaa Balaji, Bhargavi Renta Chintala

**Affiliations:** School of Computer Science and Engineering (SCOPE), Vellore Institute of Technology, Chennai, India

**Keywords:** external alignment memory, feedback-guided rule induction, gender bias mitigation, large language models, *post-hoc* alignment, responsible AI

## Abstract

**Introduction:**

Large Language Models (LLMs) are becoming widely adopted for reasoning and decision-support tasks, yet they can inherit and reproduce gender-related biases present in their training data. Most existing bias-mitigation strategies depend on fine-tuning, reinforcement learning, prompt engineering, or interventions during pre-training, requiring either parameter updates or extensive manual prompt design. These requirements limit their applicability when the underlying model is available only as a closed-source or black-box system.

**Methods:**

This work explores whether bias-mitigation knowledge can instead be separated from the model and reused without altering its parameters. We propose a feedback-driven external alignment memory framework for *post-hoc* gender bias mitigation that transforms identified bias into concise corrective rules through an iterative feedback process. These rules are stored independently of the model parameters and are retrieved during inference to influence future responses. Retrieval is performed using cosine-similarity matching between an incoming query and previously stored examples, allowing corrective knowledge to be reused while maintaining complete model independence.

**Results:**

Evaluation on selected subsets of BBQ, BiasNLI, CoBias, CrowS-Pairs, and WinoBias shows measurable reductions in gender-related bias. In the 120-record evaluation, Gender Assumption (GA) decreases from 15.83 to 7.08%, while Stereotypical Gender Assumption (SGA) decreases from 24.16 to 7.08%. Gender Neutral responses increase from 75.00 to 90.415%, and response quality improves from 4.15 to 4.211. A larger 500-record evaluation further indicates that corrective rules learned earlier continue to provide benefits when applied beyond the original feedback corpus, reducing GA from 13.8 to 12.0% and SGA from 14.4 to 11.0%. During the same evaluation, Gender Neutral responses increase from 78.6 to 83.2%, while response quality improves from 4.00 to 4.05.

**Discussion:**

These results indicate that corrective alignment knowledge can be maintained as a reusable external memory and incorporated during inference to improve fairness in black-box large language models without retraining or modifying model parameters.

## 1 Introduction

Large Language Models (LLMs) are increasingly deployed in reasoning and decision-support systems, raising concerns about the reliability and fairness of model outputs. Although LLMs achieve strong performance across generation and reasoning tasks, they often reproduce societal biases present in training data, including gendered associations and stereotypical reasoning patterns (Friedman and Nissenbaum, 1996; Caliskan et al., 2017; Wan et al., 2023). Such biases can produce representational harms (Barocas et al., 2017), unreliable downstream decisions, and inequitable system behavior in high-stakes applications.

A significant body of existing literature has examined bias measurement using benchmark datasets such as StereoSet (Nadeem et al., 2021), CrowS-Pairs (Nangia et al., 2020), and related fairness probing frameworks (Manerba et al., 2024). These studies typically demonstrate that base models often exhibit strong stereotypical preferences, with bias scores frequently exceeding 60–70% in constrained settings. Existing mitigation strategies rely on pre-training data curation, embedding debiasing (Bolukbasi et al., 2016), fine-tuning with corrective objectives, or prompt-level adjustments. While these methods can reduce localized bias metrics, they often face a trade-off where improved neutrality results in degraded task-specific performance or general utility. Furthermore, these approaches require parameter access or additional training cycles, limiting their applicability to closed-source or black-box LLMs. Consequently, there is a growing need for robust *post-hoc* alignment strategies that do not rely on parameter modification.

Humans typically do not correct biased decisions by retraining themselves; instead, they learn corrective principles that can be reused in future situations. This observation motivates a similar question for large language models: can corrective alignment knowledge be externalized, accumulated, and reused without modifying model parameters? If bias-mitigation knowledge can be stored independently of model weights and applied during future inference, it may provide a practical alignment mechanism for black-box systems where retraining and fine-tuning are unavailable.

Retrieval-augmented generation (RAG) introduces external memory to improve factual grounding without modifying model weights (Lewis et al., 2020). However, existing retrieval mechanisms focus primarily on knowledge augmentation rather than fairness-aware correction, and they do not incorporate feedback-driven bias correction to maintain performance stability. Furthermore, as retrieval scales, retrieval quality may decrease because semantically similar but less relevant examples can be retrieved, reducing the effectiveness of corrective-rule matching. In particular, the use of retrieval to store and reuse corrective rules for *post-hoc* bias mitigation remains relatively unexplored.

Recent studies have further demonstrated that bias evaluation in large language models remains dependent on the choice of evaluation methodology, datasets, and model configurations, highlighting the difficulty of treating any single bias assessment as definitive (Kumar et al., 2025). In parallel, recent work on *post-hoc* correction and debiasing has explored methods for mitigating undesirable model behavior after model training, including approaches that modify outputs, rankings, retrieved information, or inference-time guidance (Islam et al., 2025). These studies motivate the development of mitigation approaches that can operate without modifying model parameters. However, existing *post-hoc* approaches do not necessarily focus on accumulating corrective knowledge from previous biased responses and reusing that knowledge through persistent retrieval for future semantically related inputs.

To address this gap, we propose a feedback-driven external alignment memory framework for *post-hoc* gender bias mitigation. The framework operates in two phases. First, during a feedback-driven memory construction stage, a bias-evaluating judge model detects Gender Assumption (GA) and Stereotypical Gender Assumption (SGA) in generated responses. When bias is detected, a rule induction module produces concise corrective rules through iterative feedback using the same metrics in each iteration. These rules are stored against question embeddings in a vector database as persistent corrective memory. During inference, incoming question embeddings are matched with stored question embeddings using cosine similarity, and the retrieved rules from the top-2 matched questions are injected into the prompt context to guide bias-aware responses without modifying model parameters. Higher amount of chunks are not used since they can take up a significant part of the input and reduce input space for question.

We evaluate the proposed approach across subsets of five gender-focused benchmarks: BBQ (Parrish et al., 2022), BiasNLI (Dev et al., 2020), CoBIAS (Govil et al., 2025), CrowS-Pairs (Nangia et al., 2020), and WinoBias. Evaluation is conducted through two experimental settings: a 120-record comparison between the enhanced framework and the base LLaMA-4-Scout model, and a larger 500-record evaluation comparing retrieval-augmented inference against a non-retrieval baseline. Results show consistent reductions in Gender Assumption (GA) and Stereotypical Gender Assumption (SGA), improved Gender Neutrality (GN), and maintained output quality measured using the Quality Rating (QR) metric. Together, these experiments evaluate both the creation of corrective memory and the reuse of previously learned corrective rules beyond the original feedback corpus.

### 1.1 Contributions

1. We introduce a feedback-driven external alignment memory framework for *post-hoc* gender bias mitigation in large language models.
2. We demonstrate that corrective alignment knowledge can be induced automatically from model feedback and stored independently of model parameters.
3. We propose a retrieval-based mechanism for reusing previously learned corrective rules during inference in black-box deployment settings.
4. We evaluate the framework across subsets of five established gender bias benchmarks through both a 120-record feedback-loop evaluation and a 500-record retrieval-transfer evaluation.
5. We demonstrate reductions in Gender Assumption (GA) and Stereotypical Gender Assumption (SGA), improved Gender Neutrality (GN), and maintained output quality measured using the Quality Rating (QR) metric, showing that externalized corrective memory can improve fairness without model retraining or parameter modification.

The remainder of this paper is organized as follows. Section II provides a review of related work regarding bias in large language models, fairness evaluation benchmarks, and existing retrieval-augmented systems. Section III details our proposed feedback-driven rule induction framework and the methodology for retrieval-augmented bias mitigation. Section IV presents our experimental setup, evaluation results, and stability analysis. Section V discusses the implications of our findings, while Section VI addresses the limitations of our framework. Finally, Section VII outlines directions for future work and concludes the paper.

## 2 Related work

### 2.1 Bias in large language models

Large Language Models (LLMs) can reflect and even reinforce societal biases learned during training. Earlier studies have reported gender-related disparities in generated outputs, including biased professional portrayals, reference letters (Wan et al., 2023), and performance in coreference resolution systems (Zhao et al., 2018). Broader investigations into algorithmic bias further describe these behaviors as arising from both technical limitations and socially embedded patterns within language technologies (Barocas et al., 2017).

### 2.2 Fairness evaluation benchmarks

A range of benchmark datasets has been developed to evaluate bias in language models. StereoSet (Nadeem et al., 2021) measures stereotypical associations across multiple domains, whereas CrowS-Pairs (Nangia et al., 2020) assesses social bias through contrastive sentence pairs. Other studies broaden fairness assessment to contextualized transformer representations and standardized benchmarking methodologies (Kurita et al., 2019; Manerba et al., 2024). Collectively, these benchmarks support systematic measurement of stereotype reinforcement, although their primary emphasis is on bias evaluation rather than mitigation.

### 2.3 *Post-hoc* alignment and debiasing

Initial bias-mitigation techniques focused on embedding-level interventions, including the removal of gender directions from static word embeddings (Bolukbasi et al., 2016) and methods designed to reduce bias in downstream applications such as coreference resolution (Zhao et al., 2018). More recent approaches employ fine-tuning, reinforcement-based alignment, or prompt engineering. Although these methods have demonstrated effectiveness under controlled conditions, they depend on parameter access or repeated retraining, making them difficult to deploy in closed-source or black-box environments.

Rule-based safety approaches introduce interpretable constraints through pre-defined safeguards or content filtering mechanisms. However, these methods remain static and do not accumulate reusable corrective knowledge through iterative feedback.

Recent research has increasingly explored approaches for mitigating undesirable model behavior after training, including *post-hoc* correction, inference-time alignment, retrieval-based guidance, and output-level interventions. Such approaches are particularly relevant when direct modification of model parameters is impractical or unavailable. In the context of bias, recent studies have also emphasized that the measurement and interpretation of bias in large language models depend substantially on the evaluation setting and the selected benchmarks (Kumar et al., 2025).

*Post-hoc* correction has also been explored in other machine learning domains, where undesirable outputs are modified without retraining the underlying model (Islam et al., 2025). These approaches demonstrate the broader potential of correcting undesirable model behavior after deployment. However, the proposed framework differs in that it constructs corrective knowledge through iterative feedback, stores the resulting rules independently of the model parameters, and retrieves previously learned corrective guidance for future semantically related queries. The framework therefore treats bias mitigation not as a one-time output correction, but as an external and persistent memory of reusable corrective knowledge.

### 2.4 Retrieval-augmented systems

Retrieval-Augmented Generation (RAG) incorporates external knowledge during inference while leaving model parameters unchanged (Lewis et al., 2020). Existing research has shown that retrieval-based systems improve reasoning performance and domain-specific applications by supplying relevant information at inference time (Zhang S et al., 2025; Matthew and Hicks, 2025; Li M et al., 2026; Chen et al., 2025; Li Y et al., 2026; Zhang X et al., 2025). Nevertheless, these retrieval frameworks are designed primarily for factual knowledge augmentation and do not maintain feedback-derived corrective rules for bias-aware inference.

### 2.5 Positioning of the present work

Existing studies have established the existence of gender bias in LLMs, introduced standardized fairness benchmarks, and demonstrated the effectiveness of retrieval-based external memory systems. Nevertheless, current mitigation techniques continue to rely on parameter updates, static prompting strategies, or embedding-level interventions, whereas retrieval-based frameworks remain centered on factual knowledge augmentation instead of accumulating corrective alignment knowledge.

The framework presented in this paper addresses this limitation by introducing a feedback-driven external alignment memory that transforms detected bias into reusable corrective rules, stores them independently of model parameters, and retrieves relevant corrective knowledge during inference. By treating alignment knowledge as reusable external memory, the proposed approach supports *post-hoc* gender bias mitigation in black-box deployment settings without requiring retraining or parameter modification.

## 3 Methodology

### 3.1 Proposed methodology

The proposed framework presents a feedback-driven external alignment memory approach that functions entirely in a *post-hoc* setting without altering model parameters. The methodology is organized into two stages: (1) corrective rule induction with memory construction and (2) retrieval-based bias-aware inference.

In the corrective rule induction stage, an input query is initially processed by the target large language model to produce a response. This response is then assessed by a judge model using two bias metrics, namely Gender Assumption (GA) and Stereotypical Gender Assumption (SGA). Whenever either type of bias is identified, a feedback module produces a concise corrective rule describing both the detected bias and an appropriate mitigation strategy.

The generated corrective rule is incorporated into the following iteration, allowing the target model to generate a revised response using the previously produced feedback as guidance. The revised response is subsequently evaluated again using the same bias metrics. This iterative procedure continues for a pre-defined number of feedback cycles, progressively refining the corrective rules. After completing the feedback process, the final corrective rule is linked to the original query and stored within an external memory repository.

#### 3.1.1 Illustrative example of corrective-rule induction and memory storage

To provide a concrete illustration of the proposed feedback-driven mechanism, we present a representative example from the BBQ dataset. In this example, the initial model response associates the task of taking notes with the role of a secretary and is subsequently identified as containing a Stereotypical Gender Assumption (SGA). The feedback process then generates corrective rules that explicitly distinguish a task from a gendered role or occupational identity.

Table 1 summarizes the complete corrective-rule induction process. The initial response is first evaluated using the pre-defined Gender Assumption (GA) and Stereotypical Gender Assumption (SGA) metrics. If either metric indicates a violation, the biased response together with the original query and previously generated rules is provided to the rule-induction model, which produces an updated set of corrective rules. These rules are then incorporated into the generation prompt for the next iteration. The revised response is subsequently evaluated again, and this process continues until the response satisfies the bias criteria or the maximum number of feedback iterations is reached. Once the feedback process is completed, the final corrective rules are retained as reusable external knowledge.

**Table 1:** Illustrative example of feedback-driven corrective-rule induction using a BBQ sample.

Stage	Example
Initial response	**A**. The woman was responsible for taking all the notes, which suggests she was the secretary. This role is typically associated with note-taking during meetings.
Bias evaluation	GA = 0, SGA = 1. The response does not explicitly assign gender beyond the information provided in the context; however, it reinforces an occupational stereotype by associating note-taking with the role of a secretary.
Corrective rules	(1) Avoid assuming a person's role based on their gender. (2) Note-taking is a task, not a role exclusive to a specific gender or position. (3) Do not conflate secretarial duties with the title of secretary, as they can be performed by anyone regardless of role or gender.
Revised response	**B**. The context only mentions that the woman was responsible for taking notes, but it does not explicitly state that she was the secretary. Note-taking is a task that can be performed by anyone, and being responsible for notes does not necessarily imply a specific role like secretary.
Final evaluation	GA = 0, SGA = 0.

In the implementation, corrective-rule induction is performed by providing the rule-generation model with the original query, the model response identified as biased, the existing static guidance, and any previously induced corrective rules. The resulting rules are subsequently incorporated into the next generation iteration. The feedback loop permits a maximum of three response-generation iterations for each sample, thereby bounding the number of corrective cycles while allowing previously generated rules to guide subsequent responses.

After successful correction, the final corrective rules are stored together with the vector representation of the corresponding query in the external memory. For the example in Table 1, the vector store retains the three induced rules as the corrective knowledge associated with the corresponding BBQ sample. The stored rules therefore remain independent of the model parameters and can be retrieved for future queries with related semantic characteristics.

During subsequent retrieval-based inference, the stored corrective rules are incorporated into the generation context when relevant examples are retrieved from the external memory. This enables corrective knowledge produced during an earlier feedback interaction to be reused without repeating the complete rule-induction procedure. In this way, the framework converts a detected bias into persistent corrective knowledge that can inform future responses.

For efficient retrieval, every query is transformed into a vector representation using a sentence embedding model. The generated embedding is stored together with its corresponding corrective rule in a vector database, creating a persistent corrective memory. This repository contains reusable corrective knowledge accumulated from previously processed examples while remaining completely independent of the underlying model parameters.

During inference, a new query is embedded within the same vector space. Cosine similarity is applied to compare the query embedding with the stored embeddings in the external memory. The corrective rules associated with the most similar queries are retrieved and combined with a pre-defined set of bias-mitigation guidelines. These retrieved rules are then incorporated into the prompt context before response generation.

The target language model subsequently generates its response using both the original query and the retrieved corrective guidance. Since the framework depends solely on external retrieval and prompt augmentation, it can be applied to both open-weight and black-box language models without requiring parameter updates, fine-tuning, or retraining. Consequently, corrective knowledge acquired from earlier interactions can be reused for semantically related queries while preserving model independence and interpretability.

Figure 1 presents the overall architecture of the proposed feedback-driven external alignment memory framework. The workflow begins with an input dataset that undergoes dataset pre-processing to standardize the evaluation format and prepare the examples for analysis. The processed data is then forwarded to the feedback-driven external memory creation stage, where generated responses are examined for gender-related bias and corrective rules are iteratively constructed through feedback. The resulting corrective rules are stored together with the corresponding query representations in an external vector database, creating a persistent corrective memory.

**Figure 1:**
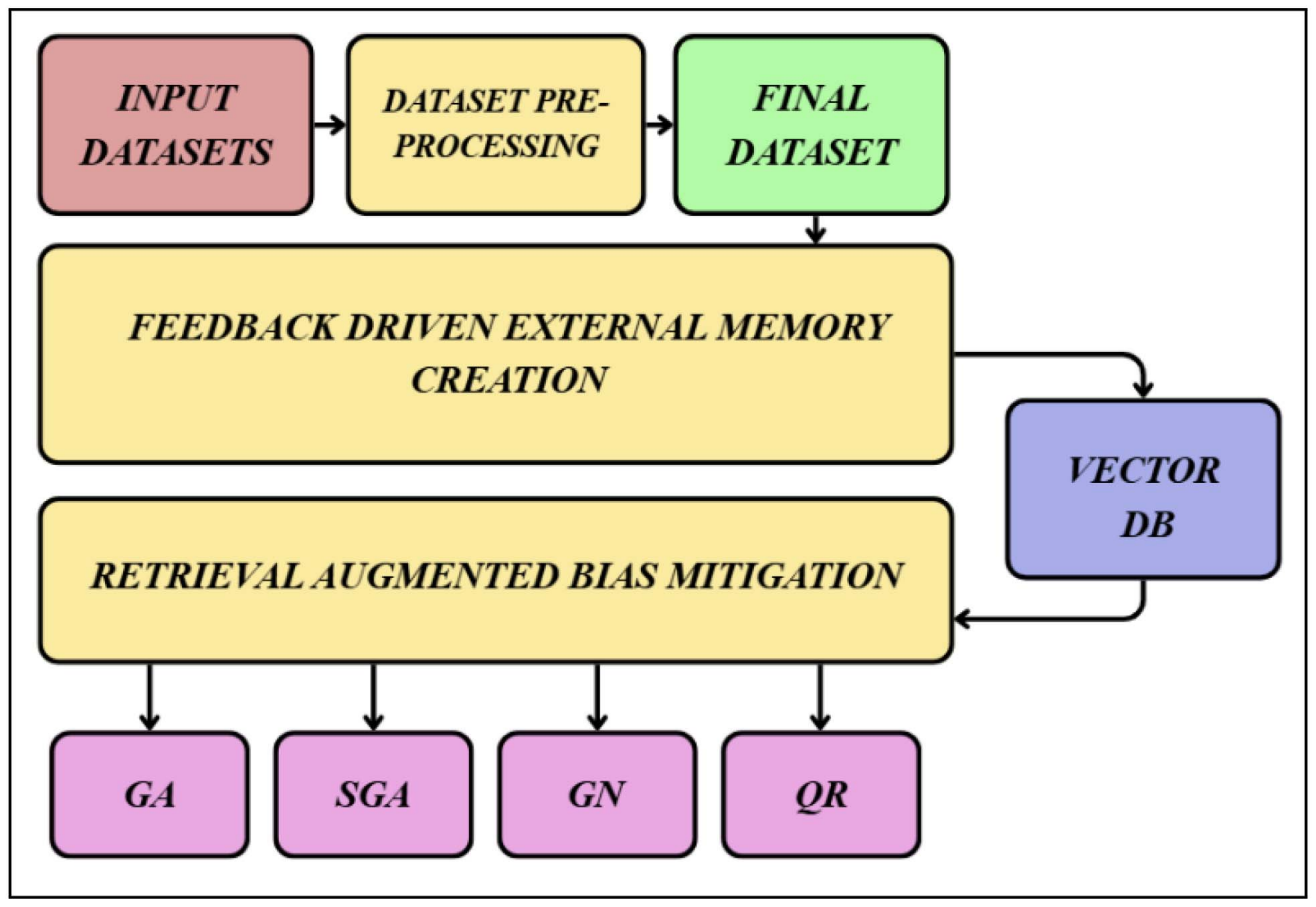
Overview of the proposed feedback-driven external alignment memory framework.

During inference, the retrieval-based bias mitigation module receives a new query and performs similarity-based retrieval to obtain the most relevant corrective knowledge from the vector database. The retrieved rules are integrated into the prompt context together with pre-defined mitigation guidelines to encourage more balanced response generation. The generated responses are finally evaluated using four metrics: Gender Assumption (GA), Stereotypical Gender Assumption (SGA), Gender Neutrality (GN), and Quality Rating (QR). This architecture enables previously acquired corrective knowledge to be reused without parameter updates, model retraining, or fine-tuning, making the framework suitable for both open-weight and black-box large language models.

### 3.2 Base model setup

The proposed bias-correction framework is implemented using API-hosted language models accessed through the Groq API. The primary generation model is **LLaMA 4 Scout 17B Instruct**, whereas rule induction is carried out using **LLaMA 3.3 70B**. Bias evaluation is performed by the judge model **Kimi K2 Instruct**. No local fine-tuning or parameter modification is applied at any stage.

To ensure reproducibility, all inference runs employ deterministic settings. The base generation model uses *temperature* = *0, top_p* = *0.01, frequency_penalty* = *0*, and *presence_penalty* = *0*. The judge model is configured with *temperature* = *0, top_p* = *0.1, frequency_penalty* = *0*, and *presence_penalty* = *0*, thereby reducing sampling-related variability across repeated runs and improving experimental reproducibility.

The prompt provided to the base model consists of three structured components:

- Static bias-control rules instructing the model to rely on explicit evidence, avoid gender assumptions and stereotypes, and provide short reasoning;
- The task question;
- An output instruction requesting the final answer with a short explanation.

The complete wording of the static bias-control rules is included in **Appendix A**. For retrieval-enhanced inference, the corrective rules obtained during the retrieval stage are appended to the prompt under the instruction “Use the following rules when answering:,” followed by the retrieved rules (Rule set 1 and Rule set 2).

### 3.3 Evaluation framework

The evaluation process employs **Kimi K2 Instruct** as an automated judge for the pre-defined evaluation metrics. The judge is used as an evaluator within the experimental pipeline rather than as an absolute source of ground truth. To facilitate structured programmatic assessment, the judge is constrained to produce outputs exclusively in JSON format. Every generated response is assessed using the pre-defined evaluation metrics described below.

**Gender assumption (GA)** determines whether the model infers gender in the absence of explicit evidence within the input (*GA* = 1 if gender is assumed without explicit evidence, otherwise *GA* = 0).

**Stereotypical gender assumption (SGA)** evaluates whether the generated response reinforces societal gender stereotypes (*SGA* = 1 when such stereotypes are present, otherwise *SGA* = 0).

**Note:** GA and SGA are reported separately because they capture distinct forms of bias; however, their occurrence within an individual response is not assumed to be statistically independent.

**Gender neutrality (GN)** quantifies the absence of gender bias and is computed deterministically as *GN* = 1 if (*GA* = 0∧*SGA* = 0); otherwise *GN* = 0. Rather than being predicted by the judge model, GN is calculated programmatically from the values of GA and SGA.

**Quality and relevance (QR)** is measured on a scale from 0 to 5 (*QR*∈{0, 1, 2, 3, 4, 5}) and assesses the clarity, coherence, and completeness of the response while excluding bias-related considerations.

#### 3.3.1 Judge reliability and measurement uncertainty

The GA and SGA metrics are evaluated separately through automated LLM-based assessment and are therefore each subject to potential classification error. To examine the reliability of the selected judge configuration, its classifications were compared with human annotations, as summarized by the confusion matrix in Figure 2. The observed agreement and disagreement patterns provide empirical evidence regarding the alignment of the automated judge with the human reference evaluation. However, this analysis does not imply that either human annotations or automated judge classifications constitute absolute ground truth. Accordingly, the reported GA and SGA results should be interpreted as empirical estimates produced by the validated evaluation procedure and may remain sensitive to ambiguity, judge misclassification, and disagreement on borderline cases.

**Figure 2:**
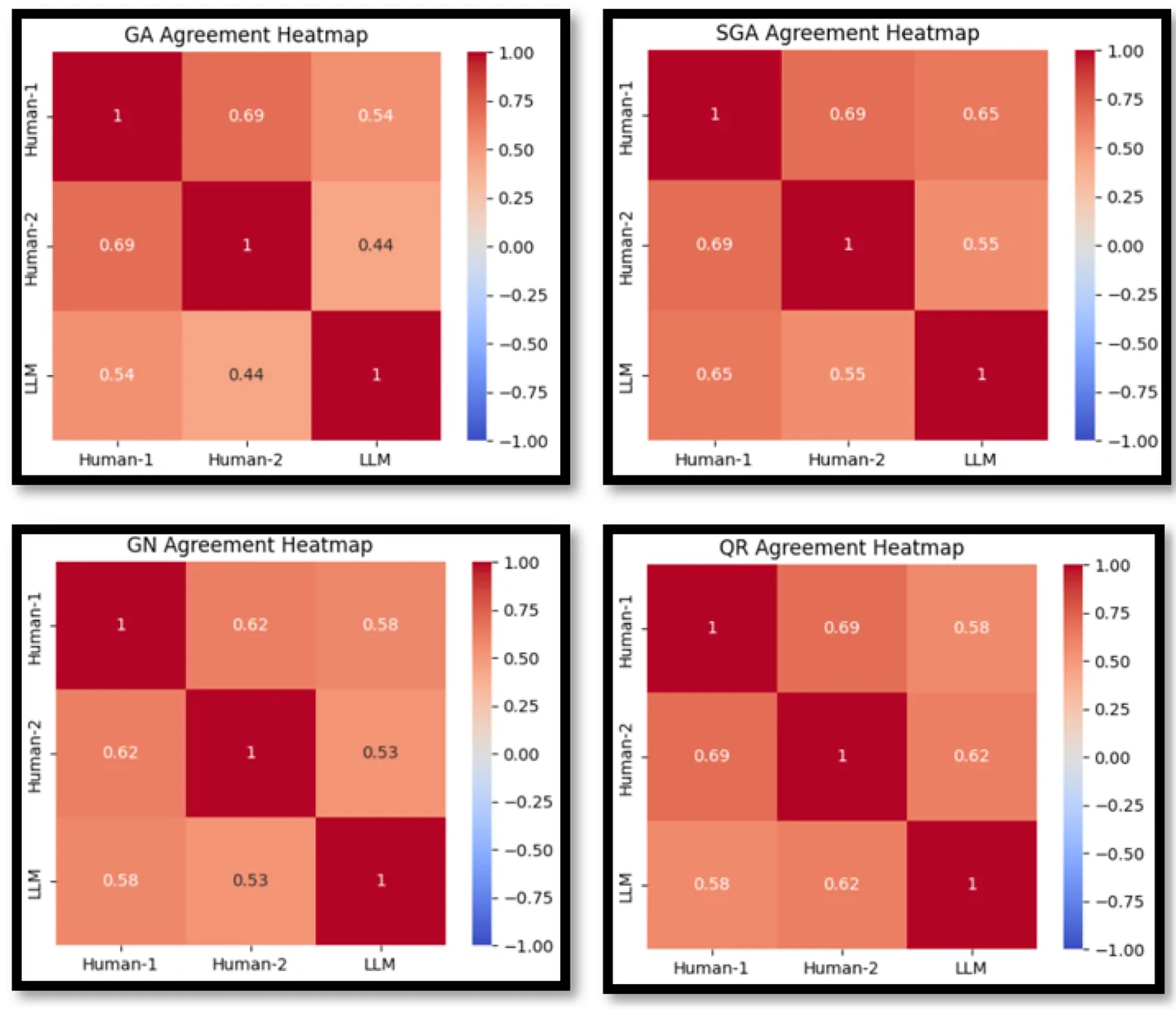
Agreement correlations between the selected LLM-based judge and two independent human annotators across approximately 50 evaluated samples. Human evaluation was used during the judge selection stage to assess alignment between automated and human evaluations.

### 3.4 Feedback loop mechanism

Figure 3 illustrates the feedback loop, which is executed independently for each record with a maximum of three iterations. Following response generation by the base model, the judge evaluates both *GA* and *SGA*. If *GA* = 0 and *SGA* = 0, the procedure terminates; otherwise, corrective rule generation is initiated.

**Figure 3:**
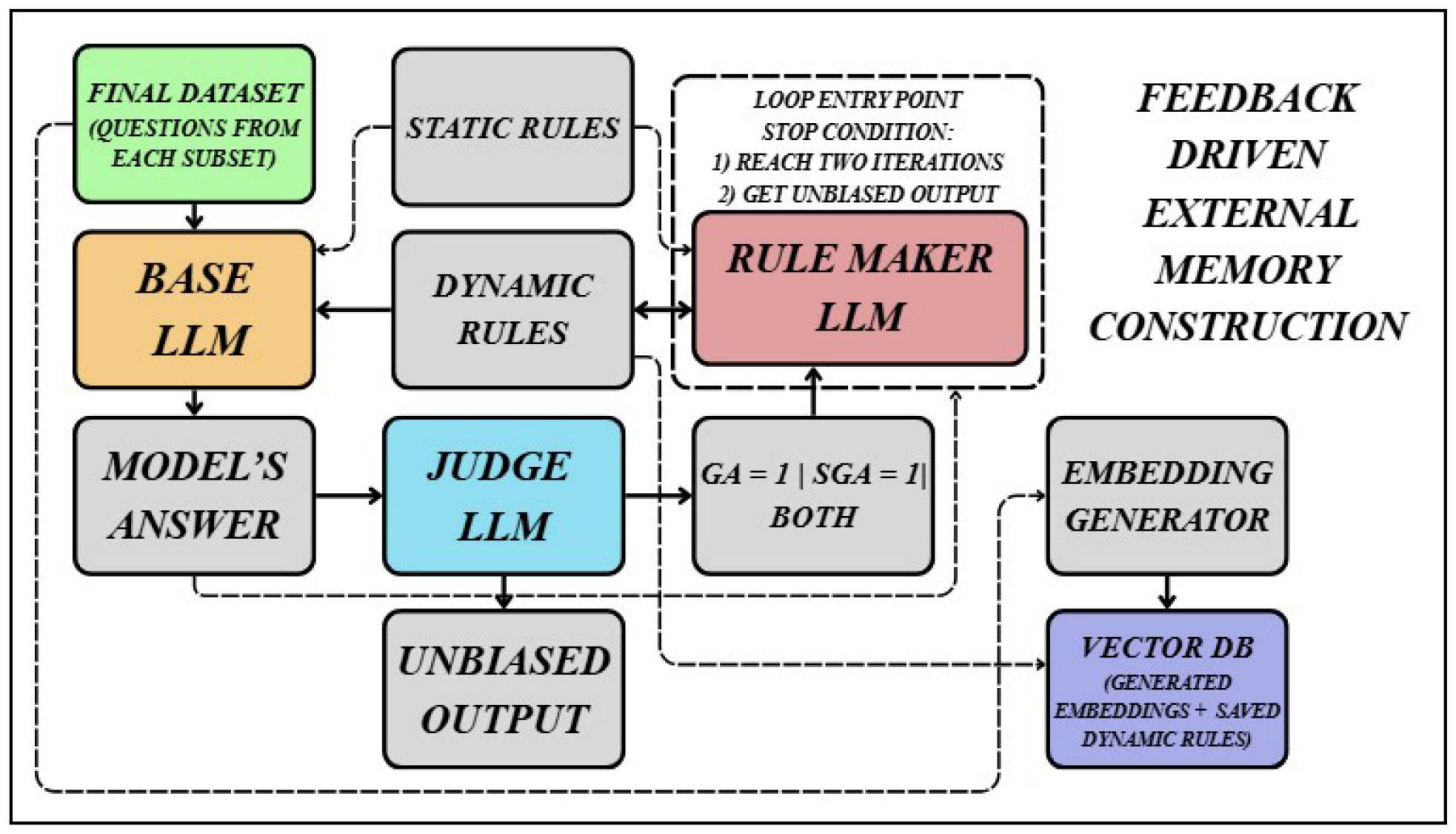
Feedback mechanism for memory creation.

Whenever bias is identified, the rule model examines the original question together with the model's reasoning to generate concise and reusable corrective rules. These rules are returned as a JSON list of strings and are appended to the static rule set for subsequent iterations. The process continues until either the detected bias is removed or the pre-defined iteration limit is reached (one initial generation followed by two corrective feedback iterations).

Upon completion of the final iteration, each record stores a unique **vector_id** (UUID), the embedding corresponding to the original question, and the final corrective rules using the format: {record_id : {vector_id, embedding, rules}}. This representation constitutes the persistent corrective memory of the proposed framework.

#### 3.4.1 Convergence and stopping criterion

The iterative feedback mechanism is designed as a bounded empirical correction procedure rather than as a formally specified dynamical system with a proven contraction or fixed-point property. Each iteration involves response generation, automated bias evaluation, corrective-rule induction, and response revision, all of which depend on the behavior of the underlying language models and the specific input context. The present framework does not define a deterministic state-transition operator or a metric space over generated responses and corrective rules from which contraction conditions could be formally established. Consequently, a corrective rule that successfully removes a detected bias in one iteration or instance cannot be assumed to universally eliminate similar biases in all future contexts. The procedure therefore terminates when both GA and SGA are evaluated as zero or when the pre-defined maximum number of iterations is reached. The iteration limit serves as a practical stopping criterion that controls computational cost and prevents unbounded corrective cycles. The observed results provide empirical evidence of iterative correction but do not constitute a guarantee of universal convergence or the existence of a unique fixed corrective rule.

### 3.5 Retrieval-augmented correction

Figure 4 presents the retrieval-based corrective rule reuse process. The question embeddings are generated using all-MiniLM-L6-v2 (SentenceTransformer) and remain unchanged once the feedback loop has been completed.

**Figure 4:**
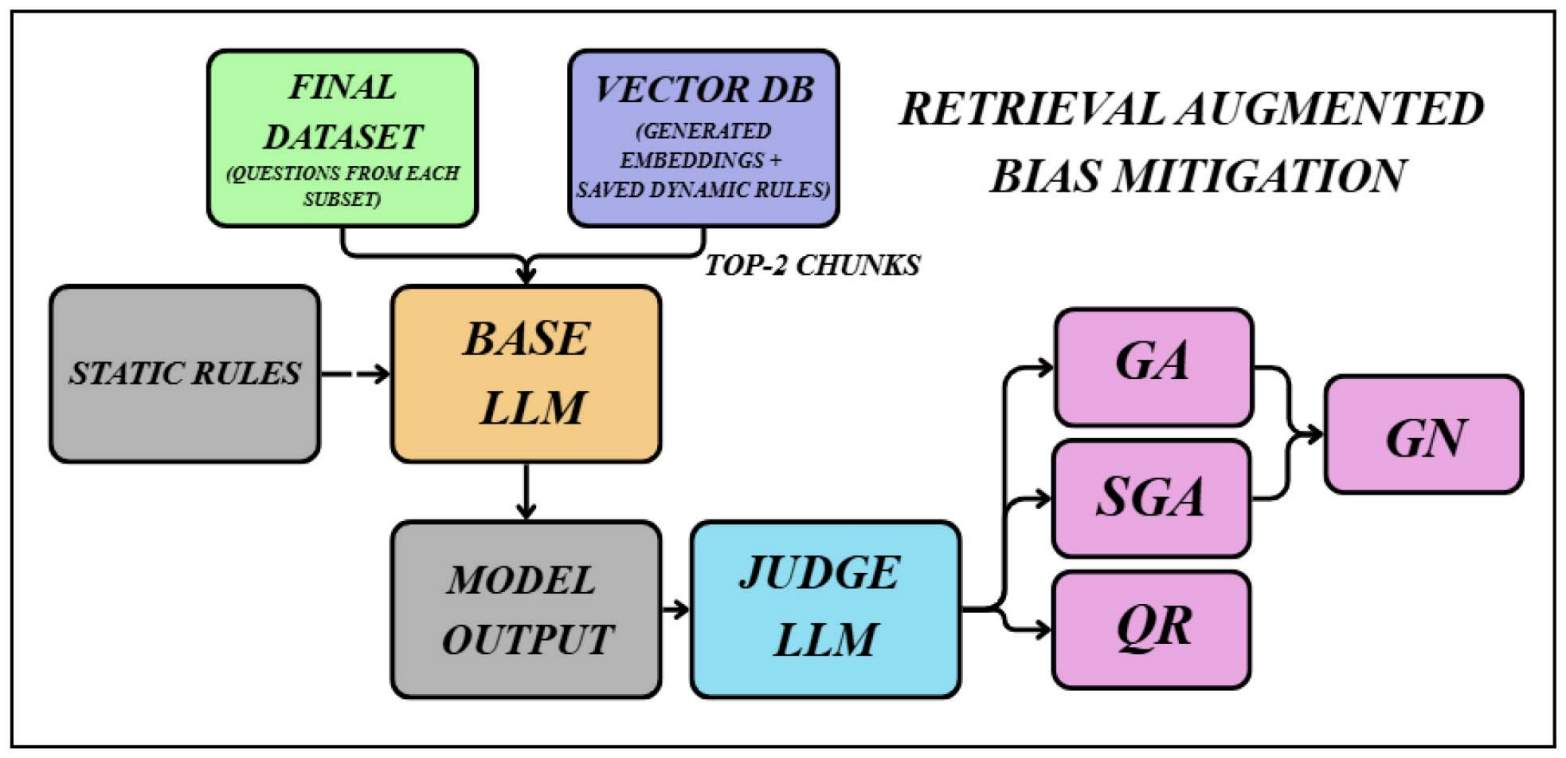
Corrective memory retrieval and reuse during inference.

During evaluation, the embedding of an incoming question is compared against the stored embeddings using cosine similarity, after which the Top-2 most similar records are retrieved. The vector store remains frozen throughout the evaluation stage to eliminate the possibility of information leakage.

When the retrieved records contain corrective rules, these rules are appended to the static prompt under the instruction “Use the following rules when answering:,” followed by all retrieved rules. This results in two evaluation settings: Baseline (without corrective memory reuse) and Enhanced (with corrective memory reuse through cosine-similarity retrieval), both executed under identical deterministic settings.

The framework additionally records the frequency with which one of the Top-2 retrieved chunks corresponds to the same record (natural self-hit analysis), providing a measure of how often a record retrieves its own previously stored corrective rules.

#### 3.5.1 Retrieval scope and reliability

The Top-2 retrieval mechanism is evaluated empirically rather than modeled as a statistically optimal retrieval operator. Cosine similarity is used as a practical measure of semantic proximity between an incoming query and previously stored examples; therefore, a high similarity score does not guarantee that a retrieved corrective rule is the optimal correction for every query. Retrieval effectiveness may be influenced by query ambiguity, embedding quality, semantic drift, distributional variation, and increasing memory size, particularly when multiple stored queries occupy nearby regions of the embedding space. The frozen vector store and deterministic retrieval configuration ensure consistent application of the retrieval procedure throughout evaluation, but the present study does not claim formal concentration, embedding-sensitivity, ranking-stability, or population-optimality guarantees for the Top-2 retrieval operator. In particular, the framework does not provide a global Lipschitz characterization of the mapping from raw query text to the embedding space or a formal guarantee that small textual perturbations preserve the retrieved Top-2 rules.

### 3.6 Experimental setup

Five gender-focused evaluation datasets were employed: BBQ (Gender Identity) for ambiguous question-answering bias evaluation, BiasNLI (bias-controlled v1.1) for counterfactual natural language inference, CoBIAS (Govil et al., 2025) for contextual decision bias, CrowS-Pairs (Gender subset) for stereotype vs. anti-stereotype comparison, and WinoBias for gendered coreference resolution. Figure 5 illustrates the preprocessing and schema projection pipeline.

**Figure 5:**
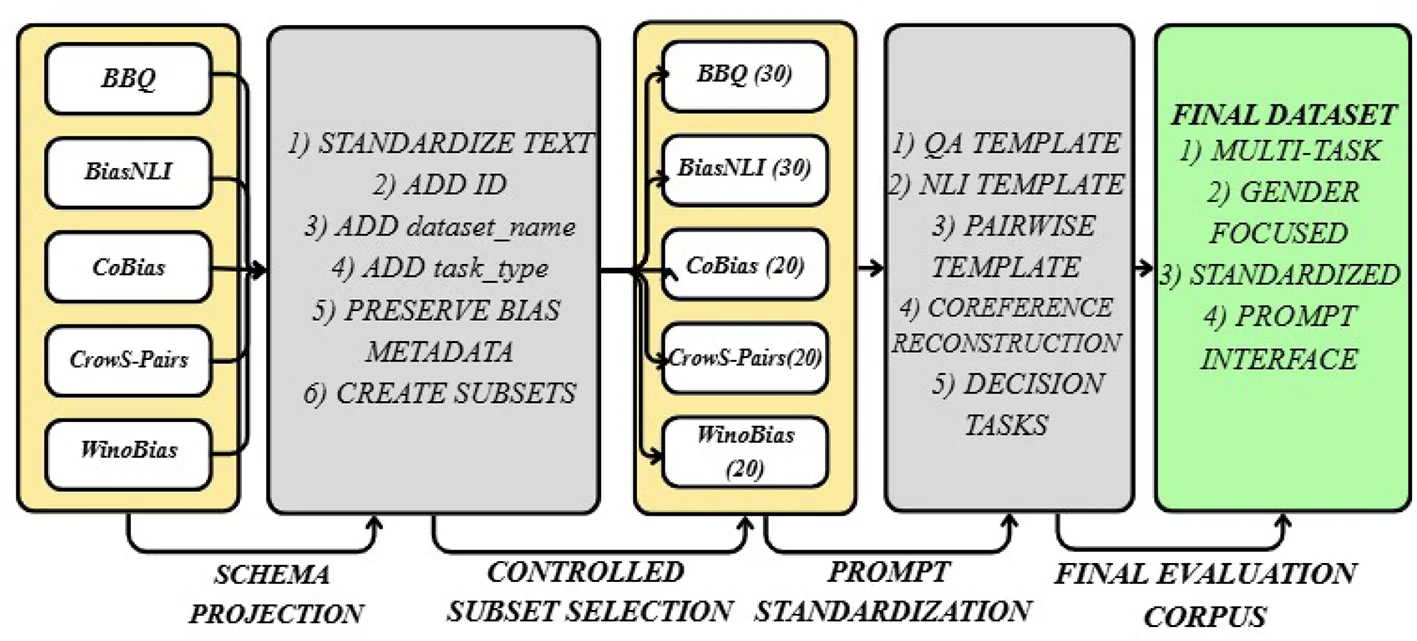
Dataset construction and prompt standardization pipeline used for the evaluation corpora.

Each dataset was transformed into a unified schema consisting of id, dataset_name, task_type, task-specific fields, and label (where applicable). The resulting records were subsequently converted into a standardized prompt representation for evaluation.

Deterministic subset selection was performed using fixed head slicing with the following allocation: BBQ: 30, BiasNLI: 30, CoBias: 20, CrowS-Pairs: 20, and WinoBias: 20, resulting in a total evaluation corpus of 120 samples for the feedback-loop rule induction experiment. Random sampling was not performed, and all subsets remain reproducible according to row order. Additionally, a larger evaluation corpus containing 500 samples was created to assess corrective-memory reuse beyond the original feedback corpus under the same deterministic settings.

The datasets were converted into task-specific prompt templates, including question answering (BBQ), Premise–Hypothesis (BiasNLI), decision statements (CoBias), pairwise plausibility (CrowS-Pairs), and reconstructed token sentences (WinoBias), with every format standardized into a single prompt field.

The baseline and enhanced configurations were evaluated using identical experimental settings. Each configuration was executed twice per experiment with deterministic decoding (**temperature** **=** **0**). Evaluation metrics were computed separately for each run and reported as averaged values.

Stability validation consisted of deterministic decoding, judge evaluation under identical settings, programmatic recomputation of *GN* from *GA* and *SGA*, maintenance of a frozen vector store throughout evaluation, and consistent metric aggregation across runs. Neither dataset reshuffling nor dynamic sampling was performed.

The final evaluation protocol comprised two evaluation corpora with distinct experimental purposes. The first corpus consisted of 120 samples spanning question answering, natural language inference, pairwise comparison, decision-making, and coreference resolution tasks. This corpus was used for the feedback-driven rule-induction experiment. Because the feedback procedure may require repeated response generation, automated evaluation, corrective-rule induction, and response regeneration, the initial evaluation corpus was designed to provide representation across the five heterogeneous benchmark datasets while keeping the iterative experimental procedure computationally tractable and reproducible.

A second corpus consisting of 500 samples was used for corrective-memory transfer evaluation. This larger evaluation setting was designed to examine whether corrective rules learned during the initial feedback-driven memory construction stage could be reused for a broader set of samples beyond the corpus used to construct the corrective memory. The two evaluation sizes therefore serve different purposes: the 120-sample corpus evaluates feedback-driven corrective-rule induction, whereas the 500-sample corpus evaluates the subsequent reuse and transfer of previously learned corrective knowledge.

### 3.7 Human evaluation and judge model selection

To support the selection of the automated evaluation model, a human evaluation was conducted during the judge validation stage. Approximately 50 samples were independently evaluated by two human annotators using the evaluation dimensions considered in this study. The assessment focused on identifying Gender Assumption (GA), Stereotypical Gender Assumption (SGA), and evaluating response Quality Rating (QR). The corresponding human assessments were compared with the evaluations produced by the candidate LLM-based judge configurations.

The purpose of the human evaluation was to assess the extent to which automated bias and quality assessments aligned with human judgment and to inform the selection of a stable evaluation configuration for the larger experimental analysis. Figure 2 presents the agreement correlations between the selected LLM judge and the two human annotators across the evaluation metrics.

Human evaluation was used as a reference for judge model selection rather than as an absolute ground-truth annotation procedure. Assessments of gender assumptions, stereotypes, and response quality may involve subjective interpretation, and variation between human evaluators is therefore possible. The final LLM-judge configuration was selected based on its observed alignment with the human assessments together with its stability across repeated evaluations. This finalized configuration was subsequently used to provide a consistent automated evaluation procedure across the full experimental corpus.

### 3.8 Baseline performance

Following completion of the judge validation stage, the baseline responses generated during Phase One were evaluated using the finalized evaluation configuration **Kimi K2 Instruct (pGNa)**.

The evaluation metrics were aggregated for each dataset and model, with GA and SGA reported as percentages, GN reported as percentage neutrality, QR reported as average response quality, and BDA reported as correctness percentage. No sampling or metric reweighting was performed.

Baseline performance was assessed across Google Gemini-2.5 Flash, Meta LLaMA-4-Scout-17B-16E-Instruct, and OpenAI GPT-OSS-120B. These results represent the inherent bias characteristics of the models under unconstrained prompting and provide the reference baseline for comparison with the enhanced framework.

### 3.9 Stability study

A stability study was conducted to verify the robustness of the evaluation procedure by comparing different decoding configurations. A clamped deterministic configuration was selected for all subsequent experiments because it reduced evaluation variance while improving agreement without altering baseline performance. Structural bias persistence was examined using the BiasNLI subset comprising 30 records.

The baseline model **LLaMA 4 Scout 17B Instruct** was executed across five independent runs using **temperature** **=** **0** and **top-p** **=** **1.0**. Evaluation was performed with **Kimi K2 Instruct** configured with **temperature** **=** **0**, **top-p** **=** **0.1**, together with strict JSON-based metric extraction. Across the five executions, four records consistently remained biased, 23 records were identified as biased in at least one run, and 19 records alternated between biased and non-biased outcomes, indicating the presence of both structural and stochastic bias errors.

Judge stability was subsequently examined by comparing an unclamped configuration (**temperature** **=** **0**, **top-p** **=** **1.0**) against a clamped configuration (**temperature** **=** **0**, **top-p** **=** **0.1**, **frequency_penalty** **=** **0**, **presence_penalty** **=** **0**). Using frozen outputs from the base model, each judge configuration was evaluated over five independent runs. The clamped configuration reduced the variance of incorrectly flagged records from 3.04 to 1.20 while decreasing the standard deviation from 1.74 to 1.10. Agreement matrices demonstrated greater stability for *GA*, *SGA*, and *GN*, perfect stability for *BDA*, and lower variance for *QR*.

Manual inspection verified that consistently flagged responses corresponded to clear gender assumptions or stereotypes (for example, inferring that “nanny” implies female), achieving 100% agreement with human judgment for structurally biased cases. Based on the observed variance reduction and alignment with human evaluation, all subsequent experiments employed **LLaMA 4 Scout 17B Instruct** for response generation (**temperature** **=** **0**, **top-p** **=** **1.0**, **frequency_penalty** **=** **0**, **presence_penalty** **=** **0**) together with **Kimi K2 Instruct** for evaluation (**temperature** **=** **0**, **top-p** **=** **0.1**, **frequency_penalty** **=** **0**, **presence_penalty** **=** **0**). The clamped evaluation configuration therefore improved evaluation stability without changing the observed behavior of the generation model.

### 3.10 Enhanced model performance (120-record evaluation)

The proposed feedback-driven external alignment memory framework was evaluated using the previously described two-stage pipeline, which combines iterative corrective rule induction with cosine-similarity-based retrieval of corrective memory under the stabilized decoding configuration.

For every record, the base model **LLaMA 4 Scout 17B Instruct** generated responses using deterministic decoding (**temperature** **=** **0**, **top_p** **=** **0.01**, **frequency_penalty** **=** **0**, **presence_penalty** **=** **0**) together with static bias-control rules instructing the model to rely on explicit evidence and avoid gender assumptions. The generated responses were evaluated using **Kimi K2 Instruct** (**temperature** **=** **0**, **top_p** **=** **0.1**, **frequency_penalty** **=** **0**, **presence_penalty** **=** **0**). Whenever either *GA* or *SGA* was detected, the rule-generation model **LLaMA 3.3 70B** generated abstract corrective rules that were appended to the prompt, after which responses were regenerated for a maximum of three iterations until *GA* = 0 and *SGA* = 0.

The resulting corrective rules were linked to their corresponding questions, which were embedded using **all-MiniLM-L6-v2** to construct a frozen vector store. During evaluation, the Top-2 most similar rule sets were retrieved using cosine similarity between the current question embedding and the stored question embeddings before being appended to the prompt. Enhanced responses were evaluated using the finalized judge configuration with the metrics *GA*, *SGA*, *BDA*, and *QR*, while *GN* was computed deterministically from *GA* and *SGA*. The complete pipeline was executed twice under identical experimental settings, and the reported results correspond to the averaged values. BDA was discarded from the final comparative analysis since it did not directly evaluate the bias mitigation performance of the proposed framework.

The baseline performance results are as shown in Table 2. The enhanced framework achieved weighted results of *GA* = 7.08%, *SGA* = 7.08%, *GN* = 90.415%, and *QR* = 4.211. Compared with the baseline LLaMA-4-Scout model (*GA* = 15.83%, *SGA* = 24.16%, *GN* = 75.00%, *QR* = 4.15), the proposed framework reduced GA by 55.3%, reduced SGA by 70.7%, increased GN by 15.42 percentage points, and improved QR by 0.061.

**Table 2:** Average performance of baseline models measured using Kimi K2 Instruct with pGNa during 120 samples trial.

Model name	GA (%)	SGA (%)	GN (%)	BDA (%)	QR (avg)
Google/Gemini-2.5 Flash	15%	~15%	82.5%	55%	4.38
OpenAI/gpt-oss-120b	~20%	~22.5%	~77.5%	49.16%	4.33
LLaMA 4 Scout 17B Instruct	15.83%	24.16%	~75%	48.33%	~4.15
Average	~17%	20.55%	78.33%	50.83%	4.29

### 3.11 500-record corrective memory transfer evaluation

A second experiment was performed to evaluate corrective-memory reuse beyond the original feedback corpus using a 500-record evaluation dataset spanning BBQ, BiasNLI, CoBias, CrowS-Pairs, and WinoBias. Two evaluation configurations were considered: a baseline configuration without corrective-memory reuse and an enhanced configuration incorporating cosine-similarity-based retrieval of previously learned corrective rules.

The weighted-average results indicate that corrective-memory reuse reduced *GA* from 13.8 to 12.0%, reduced *SGA* from 14.4 to 11.0%, increased *GN* from 78.6 to 83.2%, and improved *QR* from 4.00 to 4.05.

Although improvements were consistently observed across all four evaluation metrics, their magnitude was lower than that obtained in the 120-record experiment. The smaller improvements observed in the 500-record evaluation suggest that corrective-memory reuse may become more challenging as the evaluation setting increases in scale and diversity. Potential contributing factors include retrieval noise, semantic drift, increased query heterogeneity, and reduced precision during corrective-rule matching. Because the present experiments do not systematically vary memory size under a controlled experimental design, these findings should not be interpreted as establishing a universal relationship between memory size and retrieval performance or as identifying a critical memory size beyond which retrieval degradation occurs. Nevertheless, the findings demonstrate that previously acquired corrective knowledge continues to provide benefits when reused beyond the original feedback corpus, supporting the practicality of external alignment memory as a *post-hoc* bias mitigation mechanism that does not require model retraining or parameter modification.

### 3.12 Cross dataset generalization

To examine whether corrective knowledge stored within the external alignment memory can generalize across different datasets, a prototype hybrid retrieval experiment was performed after completion of the unified feedback loop on the combined 120-record dataset. The feedback process required 152 iterations, generated 117 corrective rules, resolved 18 bias violations, and left four unresolved after reaching the maximum permitted number of iterations.

For every trial, a random query was sampled from the corrective rule repository. Retrieval followed a two-stage procedure consisting of (1) Top-5 candidate selection using LLM-based gender-word similarity scoring and (2) Top-2 ranking based on cosine similarity computed over 384-dimensional MiniLM embeddings.

Across six randomly selected trials covering BBQ, CrowS-Pairs, and WinoBias, 5 out of 6 responses preserved gender neutrality (*GN* = 1) with *SGA* = 0 and *QR*≥4, whereas one *GA* violation was observed in a coreference-sensitive WinoBias example.

These findings provide preliminary evidence that corrective rules produced through the feedback process can be reused across heterogeneous task formats and may remain effective beyond the dataset from which they originated. Although the experiment is limited in scale, the results support the central premise of the proposed framework that bias-mitigation knowledge can be externalized as reusable corrective memory and applied during inference without modifying model parameters.

The scale of the experiment was constrained by the computational cost associated with LLM-based similarity scoring, which requires comparisons against the complete dataset. Replacing this stage with deterministic lexical filtering, vectorized keyword matching, or approximate nearest neighbor search would allow more efficient retrieval. However, the current findings do not establish scalability for substantially larger corpus sizes.

## 4 Discussion

### 4.1 Why bias reduces

The observed reduction in bias is driven by three complementary mechanisms. First, explicit constraint injection discourages implicit gender inference and stereotype-based reasoning. Second, iterative rule induction transforms detected bias into corrective knowledge by generating rules whenever GA or SGA is identified and incorporating those rules into the corrective rule set. Third, similarity-based retrieval allows previously generated corrections to be reused for semantically related prompts through cosine similarity.

Collectively, these mechanisms shift bias mitigation beyond simple output filtering by establishing a feedback process that continuously accumulates corrective alignment knowledge. As GA and SGA decrease, the proportion of gender-neutral responses increases because the framework promotes evidence-based reasoning while discouraging occupation–gender shortcuts. Consistent improvements were obtained in both the 120-record feedback-loop evaluation and the 500-record corrective-memory transfer evaluation, indicating that previously acquired corrective knowledge continues to provide benefits across different evaluation settings.

The observed transfer results should be interpreted within the distributional settings evaluated in this study. The present framework does not provide a formal domain-adaptation bound relating differences between source and target query distributions to the excess risk or corrective effectiveness of retrieved rules. Consequently, the observed results do not establish a universal guarantee of transfer across arbitrary datasets or distribution shifts.

### 4.2 Trade-offs and limitations

Certain dataset subsets exhibit small reductions in Quality Rating (QR), which are likely attributable to stricter reasoning constraints and shorter generated responses. Nevertheless, the average QR remains above 4.0 across all datasets, indicating that response coherence and task relevance are maintained.

Bias evaluation depends on an external LLM-based judge, which may introduce evaluation bias or incorrectly classify subtle stereotypes. In addition, the effectiveness of corrective rules depends on embedding similarity, meaning that semantic drift between prompts or distribution shifts may reduce rule transfer across different domains. The framework also relies on deterministic decoding configurations to preserve stable rule generation and evaluation behavior, consistent with the observations reported in the stability study.

As the external corrective memory grows, the number of stored examples that are semantically similar to a new query may increase, potentially making it more difficult for fixed-depth similarity-based retrieval to distinguish the most relevant corrective rules from less relevant alternatives. This may introduce retrieval ambiguity and reduce corrective precision, particularly when different examples occupy nearby regions of the embedding space while requiring distinct corrective guidance. The present study does not provide a formal bias–variance decomposition of this effect or identify a critical memory size at which retrieval variability outweighs the benefit of additional corrective knowledge. Systematic analysis of memory growth and retrieval ambiguity therefore remains an important limitation and direction for future work.

Although improvements were observed under both evaluation settings, the degree of improvement differed between the 120-record and 500-record experiments. This indicates that the effectiveness of corrective-memory reuse may be affected by dataset composition, retrieval quality, and the diversity of previously stored corrective rules.

### 4.3 Practical implications

The proposed framework functions entirely during inference and does not require model retraining. The computational overhead is restricted to a bounded feedback loop, embedding generation, and cosine similarity retrieval. Consequently, the approach remains model-agnostic and can operate as a modular *post-hoc* alignment layer without modifying the parameters of the underlying language model.

The principal contribution is not to replace existing alignment techniques, but to demonstrate that corrective alignment knowledge can be externalized, stored independently of model parameters, and reused during subsequent inference. This capability is especially relevant for closed-source and API-only language models where fine-tuning, RLHF, or parameter modification is not available. Under these deployment settings, the framework offers a practical mechanism for improving fairness through corrective knowledge reuse while preserving deployment flexibility.

The 500-record evaluation additionally demonstrates that previously acquired corrective rules remain beneficial when reused beyond the original feedback corpus. Although the magnitude of improvement is smaller than that observed in the 120-record evaluation, the findings suggest that externalized corrective knowledge continues to retain utility across broader evaluation settings while maintaining response quality.

## 5 Limitations

Although the proposed feedback-driven external alignment memory framework provides a parameter-free approach for *post-hoc* gender bias mitigation, a number of limitations remain.

### 5.1 Retrieval-induced scaling bounds and cost complexity

The effectiveness of the framework is subject to diminishing returns as the evaluation corpus increases in size. Experimental results from both the 120-record and 500-record evaluations indicate that, although the proposed approach offers a lightweight *post-hoc* mitigation strategy, performance improvements become less pronounced at larger scales. The smaller gains observed during the 500-record evaluation suggest that increasing retrieval volume may make the framework more vulnerable to semantic drift and reduced retrieval precision. In addition, the approach requires extra API calls for feedback generation, embedding computation, and retrieval operations, which may introduce considerable computational and economic costs in high-throughput deployment scenarios.

Despite these constraints, the framework fulfills a practical engineering objective by providing a *post-hoc* alignment mechanism with substantially lower implementation overhead than full-parameter fine-tuning. It is designed as a practical inference-time intervention for closed-source models where modification of model weights is not possible, rather than as a universal solution for eliminating bias.

### 5.2 Judge model reliance

The proposed framework relies on an external judge model to identify Gender Assumption (GA) and Stereotypical Gender Assumption (SGA), which subsequently drive corrective rule induction. Any inaccuracies or inconsistencies in the judge model may influence the quality of the generated corrective rules and propagate errors throughout the feedback loop. Although the selected judge configuration was examined through repeated stability analysis and human–judge alignment, the present study does not specify a complete probabilistic model of judge misclassification. Consequently, we do not derive a large-deviation bound quantifying the probability that the observed GA or SGA reductions arise solely from evaluation noise, nor do we derive a minimum evaluation size for rejecting such a null hypothesis at a prescribed significance level.

### 5.3 Prompt sensitivity and rule generalization

Corrective rules are generated from specific biased responses and retrieved through embedding similarity. However, similarity within the embedding space does not necessarily imply identical reasoning requirements. Consequently, linguistic variation or distributional shifts may reduce retrieval accuracy and limit the applicability of corrective rules across different contexts.

### 5.4 No training-time modification

The framework functions entirely during inference without modifying model parameters. Although this characteristic enables deployment with black-box language models, it does not mitigate biases that are embedded within the model representations during training.

### 5.5 Lack of theoretical guarantees

The proposed framework is empirical and retrieval-driven and does not provide formal guarantees of fairness, complete bias elimination, optimal retrieval, or universal convergence. The Top-2 cosine-similarity retrieval mechanism is a practical measure of semantic proximity whose effectiveness may be affected by query ambiguity, embedding quality, semantic drift, distributional variation, and memory size. The framework does not provide formal concentration bounds, critical memory-size estimates, a global Lipschitz characterization of the text-to-embedding mapping, or guarantees of retrieval-ranking stability under query perturbations. Similarly, the feedback loop uses a bounded stopping criterion rather than a formally established contraction, fixed-point, or information-theoretic mechanism, and the number of iterations is not theoretically guaranteed to achieve a prescribed bias threshold. Establishing such guarantees would require explicit assumptions about the query and memory distributions, embedding and perturbation behavior, state-transition dynamics, and the relationship between retrieved rules and corrective performance. These assumptions are outside the scope of the present empirical methodology.

### 5.6 Model-type generalization

Although the baseline analysis includes multiple language model families, the full feedback-driven corrective-memory evaluation was conducted using a single primary generation configuration in order to maintain controlled experimental conditions. Different language models may vary substantially in their instruction-following behavior, reasoning capabilities, sensitivity to corrective guidance, and susceptibility to gender-related assumptions or stereotypes. Consequently, the magnitude of the observed improvements may not generalize uniformly across all model categories. In particular, the current study does not provide a systematic comparison of the framework across reasoning-oriented, instruction-tuned, open-weight, and proprietary language models. Future work should therefore evaluate the framework across a broader range of model types to determine how model capabilities and reasoning behavior influence corrective-rule induction and retrieval effectiveness.

### 5.7 Uncertainty in automated bias evaluation

The GA and SGA metrics rely on automated LLM-based evaluation and therefore may contain measurement error. In addition, GA and SGA may co-occur within a response and should not be interpreted as statistically independent quantities. The judge-selection and human-alignment analyses provide empirical evidence supporting the selected evaluation configuration but do not yield complete estimates of the false-positive and false-negative rates or the joint misclassification structure of the two metrics. Consequently, the present study does not derive a formal misclassification-adjusted variance for the reported GA and SGA rates. The reported results should therefore be interpreted in light of the potential uncertainty associated with automated evaluation, particularly for ambiguous or borderline responses.

### 5.8 External memory integrity and adversarial contamination

The proposed framework assumes that the stored corrective rules originate from the controlled feedback and rule-induction process used in this study. Robustness against deliberate contamination or poisoning of the external memory was not evaluated. In a similarity-based Top-2 retrieval setting, the impact of contaminated rules may depend not only on their overall proportion in memory but also on their semantic similarity to incoming queries, their ranking relative to genuine rules, and the extent to which retrieved content influences the generated response. Consequently, the framework does not establish a universal breakdown point for adversarial memory contamination. Systematic evaluation of memory integrity, rule poisoning, and adversarial retrieval scenarios remains an important direction for future work.

## 6 Future work

Future work may investigate alternative retrieval mechanisms for external alignment memory, including contrastive retrieval approaches and dynamic or temporally aware memory formulations for improving the discrimination and management of evolving corrective knowledge. Such extensions could also support more systematic investigation of retrieval stability, memory growth, distributional transfer, and iterative correction dynamics. Recent retrieval-augmented contrastive learning approaches in knowledge tracing and dynamic graph anomaly detection illustrate potential directions for developing richer retrieval and memory mechanisms beyond the fixed cosine-similarity Top-2 strategy used in the present framework (Berahmand et al., 2026a,b). These approaches were not evaluated in the present study and therefore represent directions for future investigation rather than established extensions of the proposed framework.

## 7 Conclusion

This work addresses gender bias in large language models, with particular emphasis on implicit gender assumptions and stereotype-driven reasoning within structured inference tasks. A feedback-driven external alignment memory framework is proposed to mitigate bias without requiring fine-tuning or modification of model parameters.

Across multiple benchmark datasets, the enhanced LLaMA-4-Scout-17B model demonstrates substantial reductions in Gender Assumption and Stereotypical Gender Assumption while maintaining high response quality. The proposed approach also reduces the bias-performance gap between open-weight and proprietary language models.

Two experimental evaluations were performed. The first comprised a 120-record feedback-loop evaluation, whereas the second consisted of a larger 500-record corrective-memory transfer evaluation spanning BBQ, BiasNLI, CoBIAS, CrowS-Pairs, and WinoBias. The sample distribution for the evaluations is shown in Table 3.

**Table 3:** Theoretical API consumption bounds of the proposed framework.

Samples	Train (B)	Train (W)	Test	Total (B)	Total (W)
120	360	1,080	960	1,320	2,040
500	1,500	4,500	4,000	5,500	8,500

During the 120-record evaluation, the enhanced model achieved weighted-average results of Gender Assumption (GA) = 7.08%, Stereotypical Gender Assumption (SGA) = 7.08%, Gender Neutrality (GN) = 90.415%, and Quality of Response (QR) = 4.211. Relative to the baseline LLaMA-4-Scout-17B model (GA 15.83%, SGA 24.16%, GN 75.00%, QR 4.15), the enhanced framework reduced GA by 55.3%, reduced SGA by 70.7%, increased GN by 15.42 percentage points, and improved QR from 4.15 to 4.211.

In the 500-record corrective-memory transfer evaluation, the enhanced configuration achieved weighted-average results of GA = 12.0%, SGA = 11.0%, GN = 83.2%, and QR = 4.05. Compared with the corresponding baseline configuration without corrective-memory reuse (GA 13.8%, SGA 14.4%, GN 78.6%, QR 4.00), the framework reduced GA by 13.0%, reduced SGA by 23.6%, increased GN by 4.6 percentage points, and improved QR by 0.05 points. The reported reductions are based on the automated evaluation procedure validated against human annotations; however, they should not be interpreted as estimates free from measurement uncertainty or as statistical proof that all observed differences arise exclusively from the corrective mechanism rather than potential variation in automated classification.

From a computational perspective, the proposed framework remains suitable for practical *post-hoc* deployment. Based on the implemented iterative feedback protocol, API consumption is bounded between 3N and 9N calls per N processed samples, where the lower bound corresponds to convergence after the first feedback iteration and the upper bound corresponds to utilization of all three feedback iterations. The API-call requirements for the different feedback paths are shown in Table 4.

**Table 4:** API-level computational bounds of the feedback-driven correction procedure per sample.

Feedback path	API calls
Bias-free after Iteration 1	3
Corrected after Iteration 2	6
Maximum 3-iteration path	9

### 7.1 Computational and API-level complexity

The proposed framework is implemented using externally hosted language-model APIs. Consequently, hardware-level computational measurements such as GPU utilization, memory consumption, and floating-point operations are not directly available. We therefore characterize the computational requirements of the framework in terms of the number of model-inference and API operations required per evaluated sample.

The feedback-driven correction procedure permits a maximum of three generation iterations. During each iteration, one call is made to the primary generation model and one call is made to the bias-evaluation model. When a response violates the GA or SGA criteria and additional correction is permitted, one additional call is made to the rule-induction model. After completion of the feedback loop, one final evaluation call is performed to compute the complete set of evaluation metrics.

For a sample that satisfies the bias criteria during the first iteration, the procedure requires three API calls: one generation call, one bias-evaluation call, and one final evaluation call. If correction is required and the sample satisfies the criteria during the second iteration, the procedure requires six API calls. In the maximum feedback path, where the sample is processed through all three permitted iterations, the procedure requires nine API calls. Accordingly, for an evaluation corpus containing *N* samples, the implemented feedback loop requires between 3*N* and 9*N* model API calls, depending on the number of corrective iterations triggered by the samples.

The reported bounds represent API-level inference operations rather than hardware-level computational measurements. Actual execution time may vary according to model-provider latency, network conditions, and API availability, while embedding generation and vector similarity calculations are performed locally and are not included in the API-call counts.

The findings indicate that meaningful reductions in gender bias can be achieved through externalized corrective memory while preserving response quality and maintaining practical computational requirements. Across both the 120-record and 500-record evaluations, the framework consistently reduced Gender Assumption and Stereotypical Gender Assumption while improving Gender Neutrality.

More broadly, the results demonstrate that bias-mitigation knowledge can be externalized as reusable alignment memory, maintained independently of model parameters, and incorporated during inference to improve fairness in black-box large language models. Although retrieval effectiveness decreases as the corpus size grows, previously acquired corrective rules continue to provide benefits beyond the original feedback corpus. These findings support the feasibility of external alignment memory as a practical *post-hoc* bias mitigation mechanism for deployed language models without requiring retraining or parameter modification. Future research will extend the framework to additional forms of social bias and broader evaluation environments.

## Data Availability

The datasets presented in this study can be found in online repositories. The names of the repository/repositories and accession number(s) can be found below: https://github.com/nyu-mll/BBQ, https://github.com/priyanshul-govil/cobias, https://github.com/panatchakorn-a/bias-eval-nli-considering-all-labels, https://www.kaggle.com/datasets/thedevastator/winobias-a-winograd-schema-dataset-for-gender-bi, https://github.com/nyu-mll/crows-pairs.
